# Strategies and tools in illumina and nanopore‐integrated metagenomic analysis of microbiome data

**DOI:** 10.1002/imt2.72

**Published:** 2023-01-09

**Authors:** Yu Xia, Xiang Li, Ziqi Wu, Cailong Nie, Zhanwen Cheng, Yuhong Sun, Lei Liu, Tong Zhang

**Affiliations:** ^1^ School of Environmental Science and Engineering, College of Engineering Southern University of Science and Technology Shenzhen China; ^2^ State Environmental Protection Key Laboratory of Integrated Surface Water‐Groundwater Pollution Control, School of Environmental Science and Engineering Southern University of Science and Technology Shenzhen China; ^3^ Guangdong Provincial Key Laboratory of Soil and Groundwater Pollution Control, School of Environmental Science and Engineering Southern University of Science and Technology Shenzhen China; ^4^ Environmental Microbiome Engineering and Biotechnology Laboratory The University of Hong Kong Hong Kong SAR China

**Keywords:** gene‐centric metagenomics, genome‐centric metagenomics, hybrid sequencing, illumina and nanopore‐integrated metagenomics, microbiome, metagenomics, nanopore sequencing

## Abstract

Metagenomic strategy serves as the foundation for the ecological exploration of novel bioresources (e.g., industrial enzymes and bioactive molecules) and biohazards (e.g., pathogens and antibiotic resistance genes) in natural and engineered microbial systems across multiple disciplines. Recent advancements in sequencing technology have fostered rapid development in the field of microbiome research where an increasing number of studies have applied both illumina short reads (SRs) and nanopore long reads (LRs) sequencing in their metagenomic workflow. However, given the high complexity of an environmental microbiome data set and the bioinformatic challenges caused by the unique features of these sequencing technologies, integrating SRs and LRs is not as straightforward as one might assume. The fast renewal of existing tools and growing diversity of new algorithms make access to this field even more difficult. Therefore, here we systematically summarized the complete workflow from DNA extraction to data processing strategies for applying illumina and nanopore‐integrated metagenomics in the investigation in environmental microbiomes. Overall, this review aims to provide a timely knowledge framework for researchers that are interested in or are struggling with the SRs and LRs integration in their metagenomic analysis. The discussions presented will facilitate improved ecological understanding of community functionalities and assembly of natural, engineered, and human microbiomes, benefiting researchers from multiple disciplines.

## Highlights

**Figure 1:**
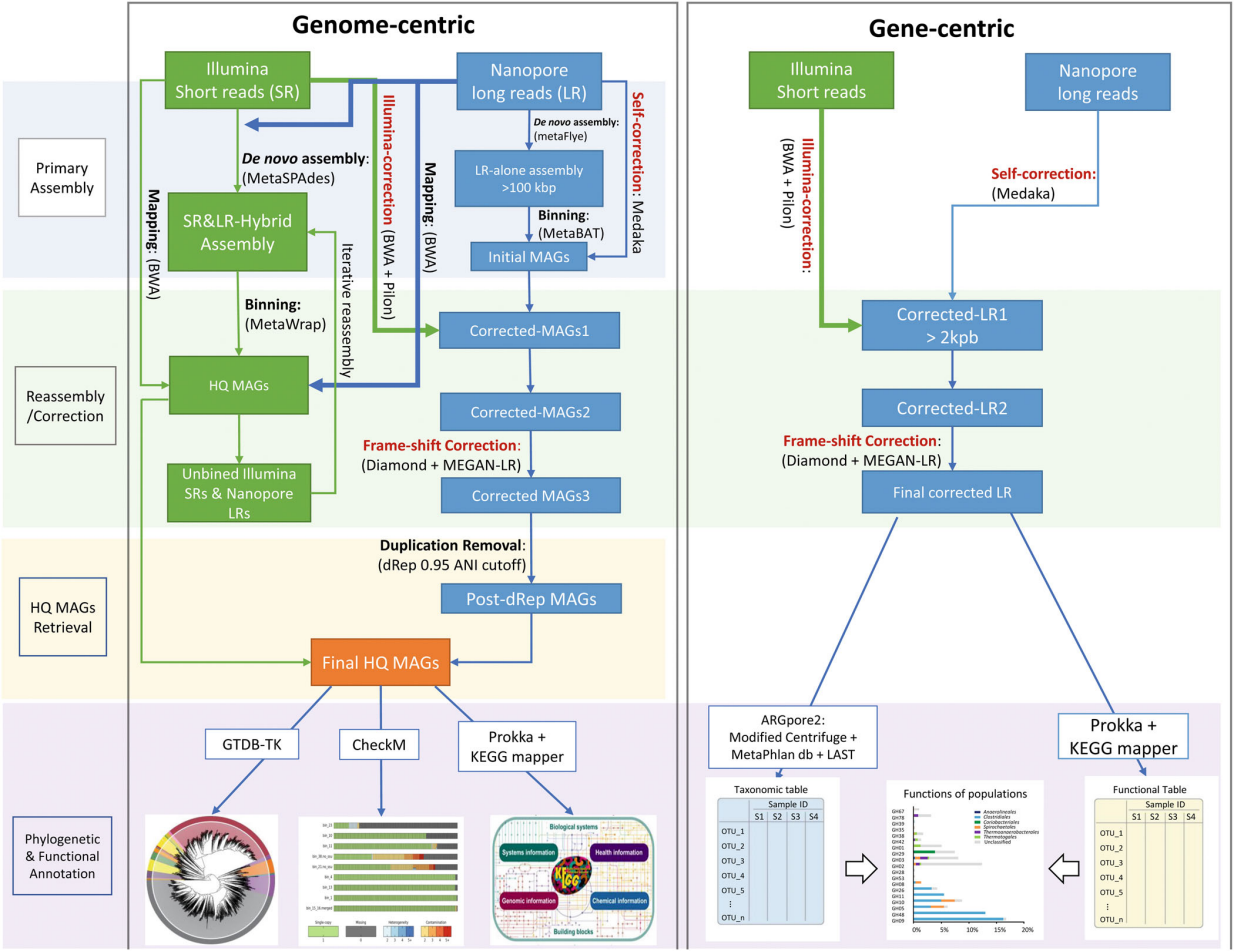
Workflow of commonly used bioinformatic strategies and tools for illumina and nanopore‐integrated metagenomic data analysis. In the “genome‐centric” analysis workflow (in the left frame), steps involved in the illumina‐orientated hybrid approach in which illumina SRs and nanopore LRs were assembled together to get primary assembly, are colored in green, while those in the nanopore‐orientated approach in which the primary assembly is derived by de novo assembly of nanopore LRs alone, are colored in blue. Integration of illumina SRs and nanopore LRs are highlighted with thicker lines. The font color of the three correction steps in the nanopore‐orientated assembly is shown in red and HQ MAGs stands for high‐quality MAGs which show completeness >90%, contamination level <5%, and with intact 16S rRNA operon [22]. The right frame illustrates the “gene‐centric” workflow with lines and annotations applied in the same manner. Bioinformatic tools commonly used in literature for each step are listed between brackets. The pictures demonstrating the output for phylogenetic and functional annotation are screen clips from the homepage of the corresponding tool. ANI, average nucleotide identity; BWA, burrows‐wheeler alignment; GTDB, genome taxonomy database; HQ, high quality; MAG, metagenome‐assembled genome; MEGAN, metagenome analyzer.

- A timely knowledge framework to integrate nanopore long reads and illumina short reads is provided.
- Workflow of common strategies for illumina and nanopore integration is illustrated in Figure 1.
- Algorithm basis and application properties of tools are summarized in Table 3.

## Graphical abstract

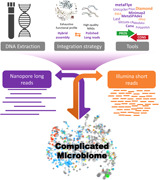

## INTRODUCTION

Studying microorganisms from a microbiome perspective is of clear merit in understanding the impact and implication of microbe‐facilitated functions and bioprocess in humans [1, 2, 3], plants [4, 5], and the natural environments [6, 7, 8, 9]. The establishment of a metagenomic whole genome (thereafter referred to as metagenomic for short) sequencing in the last decade [10, 11, 12, 13, 14] had enabled robust exploring of microbial biodiversity and functions in various natural and engineered microbiomes. However, the unevenly distributed community composition and the genome microdiversity had make the de novo metagenomic assembly of complex microbiome with illumina short reads (SRs) highly fragmented [16]. Oxford Nanopore Technology (ONT) could produce long reads (LRs) that are long enough to span most of the repetitive regions on microbe's genomes and thus significantly increase the continuity of assembly [17, 18]. In addition, LRs are able to directly span single‐nucleotide polymorphism (SNP) within a genome, enabling enhanced strain heterogeneity detection in a complex population [19]. Nevertheless, the indel errors persistent on nanopore‐assembled genome evidently hampers its applicability as a reference genome. With the design of leveraging the strength of both types of reads to address specific biological questions, a growing number of microbiome studies are combining nanopore LRs and illumina SRs in their bioinformatics analyses, termed integrated metagenomics. For example, the incorporation of nanopore LRs dramatically improved the continuity of metagenomic assemblies of human gut microbiota, which facilitated the detection of a large, expended set of structural variation (SV) types. On the basis of this integrated workflow, Chen et al. found SVs profiles are highly distinct between individuals and stable within an individual, which could be used as a gut microbiome fingerprint to present function‐associated strain‐level differentiations within gut species [20]. Even though genome assemblies of isolated strains with such hybrid approach have shown superior performance compared with either error‐prone nanopore LRs or high‐accuracy illumina SRs alone [21], the integration of these two types of reads in real metagenomic data set is not that straightforward given the existing limitations of each technology and the bioinformatic challenges associated. It is generally difficult to determine the best integration strategy (Figure 1) for a particular research purpose (the pros and cons of each strategy will be discussed in detail in subsequent sections).

Therefore, in this review, the analytical procedures of some recent milestone work implementing such integrated metagenomics were compared and summarized, to build a practical knowledge framework for readers to catch up with the latest developments in the field. As will be put forward in this review, some bold recommendations on analytical workflow were made based on the information obtained in the literature and the authors' prior experience in analyzing integrated metagenomic data sets. One final point to note for the readers is that the integrated metagenomic approach discussed here is still in the early stages of development and is subject to rapid change at the time this review is being written. Although the basic ideas underpinning the knowledge framework are reliable, it is still subject to variations imposed by bioinformatic and biotechnological developments, such as the introduction of novel assembly algorithms or the future accuracy improvements in nanopore LRs.

## ADVANTAGES AND LIMITATIONS OF ILLUMINA AND NANOPORE‐BASED METAGENOMICS IN MICROBIOME RESEARCH

The preparation of the nucleic acid target, RNA or DNA, into a form compatible with the sequencing system to be used is fundamental to illumina and nanopore library construction. The low amount of starting DNA material required for illumina library preparation, combined with the readily available commercialized illumina SR sequencing service at a low cost, has greatly facilitated its widespread application in metagenomic microbiome investigations. Although the overall cost for nanopore sequencing is still evidently higher than that of illumina sequencing at the moment, the rapid turnaround time and less restricted sequencing scenario enabled by its real‐time sequencing principle as well as the low instrumental requirements, make it irreplaceable for specific research purposes, such as tracking outbreak surveillance [23, 24, 25], on‐site microbiome profiling at remote areas [24, 26, 27], and so on. In addition, novel nanopore sequencing protocols have opened up new opportunities for microbiome research. For example, using the ReadUntil method, researchers were able to selectively eliminate the host genome sequences, resulting in precisely controlled targeted sequencing within a community [28, 29, 30, 31]. However, the current challenge for applying ReadUntil in studying the natural microbiome is the general lack of known reference genomes for decision making during selective sequencing. Recently, MetaRUpore (https://github.com/sustc-xylab/metaRUpore) has adopted a heuristic approach to circumvent this reference deficiency bottleneck and has demonstrated superior performance in retrieving near‐finish metagenome‐assembled genomes (MAGs) from the microbiome of anaerobic digesters and the human gut. Furthermore, the direct RNA protocol had enabled the direct capture of community diversity at RNA‐level [32] as well as genome sequencing of RNA viruses in their native form [33, 34]. Additionally, methylation‐calling from nanopore signals had allowed human epigenome‐wide evaluation [35]. The detailed pros and cons of illumina and nanopore‐based metagenomics in microbiome study are summarized in Table 1.

**Table 1:** Advantages and limitations of illumina and nanopore‐based metagenomics in microbiome research

	Library preparation and sequencing	Reads‐based community and functional analysis	Assembly and binning
*Illumina‐based metagenomics*			
Advantages	Readily available commercial sequencing service with a relatively low price Low requirement on the input DNA for library construction in terms of both DNA quality and quantity, for example, 1 ng DNA is enough for library construction	Massive SRs with high community coverage, easy to capture signal for populations with very low abundance Various mature bioinformatic frameworks to carry out community, functionality as well as metagenomic binning analysis	High‐accuracy SRs could ensure the accuracy of assembled MAGs
Limitations	High instrumental cost, which results in relatively longer turn‐around time to obtain sequencing data at centralized labs or sequencing companies Unavoidable biases against high‐GC populations by the bridge‐PCR	Generally difficult to assign SRs to a specific phylogenetic lineage, for example, species level	Hard to assemble exogenous elements, resulting in highly fragmented MAGs. For example, even high‐quality MAGs still have >50 contigs
*Nanopore‐based metagenomics*			
Advantages	Relatively low instrument price, which enables short turn‐around time to obtain sequencing results within 48 h at every lab Higher feasibility to customize sequencing protocols for specific sequencing purpose, for example, ReadUntil sequencing No systematic bias during sequencing, but has an evident base‐calling constrain	Long read length enables easy assignment of LRs to a specific phylogenetic lineage (e.g., species level), but correction must be applied to ensure reliable functional annotation	Outstanding capability to obtain highly continuous MAGs from metagenomic assembly
Limitations	High overall sequencing price by commercial sequencing service at present Strict requirement on DNA purity and quantity (>400 ng DNA) to ensure a successful sequencing run with expected data output	High error rates of raw LRs generated by mainstream chemistry, namely, 5%–10% for R9.4 chemistry and 3%–5% for R10.4 chemistry Regular bioinformatic pipelines, like, Prokka, MetaWRAP, unapplicable for raw nanopore‐LRs analysis	Difficulty to assemble low coverage populations due to the sequencing throughput limit which is most often associated with the high sequencing cost Persistence of indel and chimera errors on the assembled the MAGs which limited its application as reference genome

Abbreviations: GC, gas chromatography; LR, long read; MAG, metagenome‐assembled genome; PCR, polymerase chain reaction; SR, short read.

Given the strict requirements on DNA quantity and quality to ensure successful nanopore library construction, we summarized DNA extraction protocols from recent studies that had applied nanopore‐based metagenomic sequencing of environmental microbiomes in Table 2. Among the commercially available kits, DNeasy PowerSoil Kit (QIAGEN) was the most frequently used for metagenomic investigations. To ensure successful sequencing, AMPure XP beads were frequently used to clean up the shorter DNA fragments produced during extraction. Finally, one‐dimensional ligation (SQK‐LSK108 and SQK‐LSK109) emerged as the most popular sequencing protocol due to the practical trade‐off between per‐base accuracy required for bioinformatic processing and per‐flow cell data yield required to ensure adequate community coverage. Maghini et al. also reported a high‐molecular‐weight DNA extraction protocol based on enzymatic bacterial cell lysis, which could yield microgram quantities of output DNA with fragment peak lengths in the tens of kilobases from <1 g of input human stool sample [19, 46].

**Table 2:** DNA extraction protocols of recent studies applying nanopore‐based metagenomic sequencing on environmental microbiomes

Sample type	DNA extraction method	DNA purification	DNA quality and quantity	ONT library preparation	Max data yield per‐flow cell (Gbp)	Nanopore sequencing platform	Reference
Feces from infant	FastDNA Spin Kit for Soil (MP Biomedicals)	1× Hi Prep bead clean‐up	1 mg unfragmented DNA in a 46 μl volume	SQK‐LSK108	2.3	R9.5 (MIN107) MinION	[36]
				SQK‐LSK108	16.5	R9.4.1 (MIN106D) GridION	
				SQK‐LSK109	15.2	R9.4.1 (MIN106D) GridION	
Cow feces	DNA extracted by MagAttract HMW DNA Kit (Qiagen)	Qiagen DNeasy PowerSoil (Qiagen)	N.A.	SQK‐LSK108	1.6	R9.4.1 (MIN106) MinION	[37]
Stool samples	Qiagen Stool Mini kit	SPRI bead protocol	1 mg size‐selected DNA	SQK‐LSK108	27.4	R9.4.1 MinION	[22]
Sediment	FastDNA SPIN Kit (MP Biomedicals)	N.A.	N.A.	SQK‐LSK109	N.A.	R9 (MIN106D) MinION	[38]
Activated sludge from WWTP	DNeasy PowerSoil Kit (Qiagen)	SPRI bead protocol	N.A.	SQK‐LSK109	59.2	R9 (PRO002) PromethION	[39]
Activated sludge from WWTP	DNeasy PowerSoil Kit (Qiagen)	AMPure XP beads	1.5–2.0 mg of DNA	SQK‐LSK108	94.5	R9 PromethION	[18]
Zymo CS bacterial isolates	DNeasy PowerSoil Kit (Qiagen)	AMPure XP beads	1.5–2.0 mg of DNA	SQK‐LSK108	16.03	MIN106 GridION	[40]
					148.03	PRO002 PromethION	
WWTPs influent samples	DNeasy PowerSoil Kit (Qiagen)	Gel purification AMPure XP beads	1.5–2.0 mg of DNA	SQK‐LSK108	4.7	R9.4 MIN106 MinION	[41]
WWTPs effluent samples	DNeasy PowerSoil Kit (Qiagen)	Gel purification AMPure XP beads	1.5–2.0 mg of DNA	SQK‐LSK108	4.2	R9.4 MIN106 MinION	
WWTPs activated sludge samples	DNeasy PowerSoil Kit (Qiagen)	Gel purification AMPure XP beads	1.5–2.0 mg of DNA	SQK‐LSK108	5.4	R9.4 MIN106 MinION	
Adult mouse gut microbiome	QIAamp DNA Microbiome Kit (Qiagen)	N.A.	300 fmol of input DNA 25 ul	SQK‐LSK108	5.3	R9 MIN106 MinION	[42]
Groundwater	Phenol‐chloroform‐based method without mechanical lysis to minimize fragmentation	Zymo DNA Clean and Concentrator kit AMPure bead (Agencourt AMPure XP, Beckman Coulter)	DNA with a concentration of 98 ng/μl and a total amount of ~1.4 μg	SQK‐LSK109	11.58	R9.4.1 MIN106 MinION	[43]
Anaerobic sludge WWTP	DNeasy PowerSoil Kit (Qiagen)	SRE XS (Circulomics)	N.A.	SQK‐LSK109	35	R9.4.1 MIN106 MinION	[15]
				SQK‐LSK112	14	R10.4 MinION	
Soil samples from north Antarctica	DNeasy PowerSoil DNA isolation Kit (Qiagen)	N.A.	1 µg of soil DNA	SQK‐LSK109	5.7	R9 MIN106 MinION	[44]
Stool samples	QIAamp PowerFecal DNA Kit (Qiagen) TissueLyser LT (Qiagen)	N.A.	N.A.	1D Ligation protocol	N.A.	R9 MIN106 MinION	[45]

Abbreviations: 1D, one‐dimensional; HMW, high‐molecular‐weight; N.A., not available; ONT, Oxford Nanopore Technology; SPRI, solid phase reversible immobilization; WWTP, wastewater treatment plant.

## ANALYTICAL STRATEGIES FOR ILLUMINA AND NANOPORE‐INTEGRATED METAGENOMIC ANALYSIS OF MICROBIOME DATA

Likewise, to classic metagenomic analysis, there are two analytical paths for the illumina and nanopore‐integrated metagenomic data analysis: the first one is known as the “genome‐centric” approach in which genomes of different microbes within a community were separated from each other and thus got isolated into so‐called MAGs, based on coverage differences or genomic features, such as tetranucleotide frequency. The target of genome‐centric approach of illumina and nanopore‐integrated metagenomics is to obtain high‐quality MAGs (defined as estimated completeness >90%, contamination <5%, and intact 16S rRNA operon) [47] of the major populations of a community, so that the ultimate question of microbial ecology—who is doing what in the community, could be elucidated at the genome level. The other path for analyzing metagenomic data set is the assembly‐free “gene‐centric.” The target of this approach is to retrieve as much as possible the functional diversity of a community other than to achieve utmost association between functionality and specific phylotype as in the “genome‐centric” approach.

### Assembly‐based “genome‐centric” strategy

Metagenomic de novo assembly is the core step for the “genome‐centric” approach. It determines not only the performance of subsequent metagenomic binning step, but also largely the computational resources (RAM and core time) required to complete the whole analysis workflow. By far, two assembly strategies have been proposed to implement this critical assembly step, namely, hybrid‐assembly and nanopore‐assembly, respectively, shown as the green and blue parts in “genome‐centric” side of Figure 1. Just as the name implies, the illumina SRs will be assembled together with nanopore LRs in the hybrid‐assembly strategy. MetaSPAdes [48] and Unicycler [49] represent by far the most robust tools for implementing such hybrid‐assembly algorithm, in which nanopore LRs will be used to facilitate the resolution of repeats in the consensus assembly graph of illumina SRs. Worth noting is that Liu et al. reported the effectiveness of an iteratively hybrid‐assembly (IHA) in retrieving MAGs of different prevalence within a community. In the IHA method, illumina SRs and nanopore LRs included in the qualified MAGs obtained in the first round will be excluded from the second round hybrid‐assembly and binning, which could improve MAGs' recovery of the minority populations of the community by reducing sequence data complexity [18]. The first complete genome of Candidatus Brocadia reconstructed by this method revealed two identical copies of hydrazine synthase (*hzs*) genes, demonstrating genomic redundancy of this crucial phylomarker of anammox. The heavy computational requirement is the major drawback of this hybrid‐assembly strategy. Presumably owing to the high sequence complexity of the error‐prone nanopore LRs, the integration of nanopore LRs into the de novo assembly workflow of illumina SRs will rapidly take up RAM and dramatically increase the core time required to finish the analysis. Accordingly, integration of 1 Gbp of nanopore LRs with 10 Gbp illumina SRs of a permafrost microbiome data set will cause “core dump” error of the MetaSPAdes hybrid‐assembly on server with 512 Gb RAM, while assembly of 30 Gbp of illumina SRs alone could finish smoothly [50]. Therefore, it is foreseeable that for most natural environmental samples with complicated microbial communities, subsampling either random subsampling or phylogeny partition‐based subsampling [51], is unavoidable to accomplish such a hybrid‐assembly strategy for labs with regular computational resources.

To circumvent the computational limitation of the hybrid‐assembly strategy, a nanopore‐assembly‐oriented method was established. Different from hybrid‐assembly, the primary assembly of the nanopore‐assembly approach is derived by the de novo assembly of nanopore LRs dataset alone. metaFlye [16], Miniasm [52], and Canu [53] are the most popular tools for such nanopore‐alone metagenomic assembly purposes. For most metagenome data sets, Canu presented the most effective algorithm to retrieve the highest amount of the genetic information (in terms of contig size) of a community, nevertheless, the computational demand of Canu is much higher than that of the other tools. Thereafter, some researchers had suggested to conduct at least one round of self‐correction of the nanopore data sets to improve sequence accuracy as well as to reduce data size before proceeding with Canu assembly [54, 55, 56]. Additionally, the repeat‐graph simplification step of metaFlye showed a clear advantage in resolving community microdiversity by producing strain‐level genomes with large shared conservative regions [16]. Next, MAGs could be derived from the nanopore‐assembled contigs using composition or coverage‐based binning analysis. Given the invocation to obtain the complete genome profile of a microbiome, it would be a practical suggestion to take the extremely long contigs (>1 Mbp) potentially obtained from different assembly tools, directly as initial bins for subsequent binning step [13]. Afterwards, three rounds of correction steps should be conducted to improve genome reliability. Usually, the first‐round correction is the LRs self‐correction in which LRs would be aligned back to the contigs assembled to gain consensus by tools, like, Medaka or Racon [57]. The second round of correction is the step where illumina SRs get integrated into the workflow. SRs will be mapped onto the nanopore‐derived contigs to correct indel errors. Pilon [58] is the most convenient tool to identify and correct indel errors based on illumina SRs' alignment. Although minimap2 [59] showed outstanding speed in mapping large illumina SRs data set, mapping by burrows‐wheeler alignment–maximal exact matches [60] showed the higher sensitivity in identifying indels by Pilon [50]. Given the large size of the illumina dataset, this SRs‐correction step is usually the most time‐consuming step in the overall correction analysis. Additionally, Loose et al. had pointed out that Racon's consensus algorithm could further reduce indel errors on contigs corrected by Pilon, suggesting further room for improving the integration of illumina SRs [61]. The final round of correction is the frame‐shift correction step in which contigs will firstly be aligned to a comprehensive protein database, for example, NCBI Refseq protein database with frame‐shift aware DNA‐to‐protein alignment of Diamond [62] or LAST [63]. Next, based on the location of frame‐shifts reported in the alignments, Ns will be inserted into the contigs so as to maintain the frame. The community version of MEGAN6‐LR [64] could conduct such correction based on the bam file generated by Diamond, while a similar correction based on LAST alignment could be implemented by FUNpore [50]. The postcorrected MAGs could be evaluated and annotated using conventional genome quality and annotation tools, such as GTDB‐Tk [65], CheckM [66], or Prokka [67]. If multiple assemblers have been applied in your nanopore‐assembly workflow, replicated MAGs should be removed or merged by dRep [68] before annotation and quantification.

One more thing to mention is that as defined in the minimal information about a metagenome‐assembled genome standard [69], finished microbial genomes are genomes with “… a single, validated, contiguous sequence per replicon, without gaps or ambiguities” and “a consensus error rate equivalent to Q50 or better.” Even with the multiple sequencing technologies applied to pure cultures [47], this is difficult to meet this standard. Nevertheless, the second‐highest quality tier, high‐quality genome (defined as estimated completeness >90%, contamination <5%, and intact 16S rRNA operon), can be achieved despite the highly fragmented contigs by illumina SRs‐based assembly or the presence of numerous frame‐shift errors by nanopore LRs‐based assembly, both of which can have significant implications for subsequent analysis [70]. Notably, the greatest obstacle to obtain high‐quality MAGs by illumina‐based metagenomic binning analysis is the general inability to get highly continuous contigs containing intact 16S rRNA operon, which could be effectively solved by integrating nanopore LRs into the genome‐centric workflow. To fill the gap between the Q50 finished genome and the high‐quality genome, the concept of “near‐finished” genome was proposed by Sereika et al., 2022, as a high‐quality MAG for which illumina SRs polishing is not expected to significantly improve the consensus sequence [71]. And their deep sequencing of the Zymo mock community indicates that near‐finished microbial reference genomes can be obtained from nanopore sequencing with R10.4 chemistry alone at a coverage of approximately 40× [71]. However, the coverage of most species, especially the rare species, in a metagenomic data set is typically lower than the requirement of >40× coverage to reach such near‐finish consensus accuracy. Therefore, the polishing step using illumina SRs is currently critical to ensure overall quality of MAGs derived from nanopore LRs‐based assembly, highlighting the importance of the integrated metagenomic approach described in this review from a practical standpoint. With future development of adaptive nanopore sequencing or other microfluidics‐based selective enrichment techniques, adequate coverage may be reached to further improve the consensus accuracy of MAGs derived. Even with these implicit errors, the nonfragmented and well‐polished MAGs obtained from illumina and nanopore‐integrated metagenomic binning could still serve as an invaluable complement to what has already been learned about the functional capacities of the uncultivated majority of an intricate environmental microbiome. Additionally, alignment based on highly accurate PacBio HiFi reads (error rate below 1%) had showed the capacity to phase alternative SNP haplotypes to get lineage‐solved MAGs. In contrast, despite nanopore LRs had enabled reliable detection of a large and expanded set of SV types (notably including large insertions and inversions) in human gut microbiomes [20], algorithm optimization is still needed to systematically demonstrate the capability of nanopore LRs to resolve genetic variations within related populations whose genomes were initially collapsed into a single presentation during metagenomic assembly.

### Assembly‐free “gene‐centric” strategy

**Figure 2:**
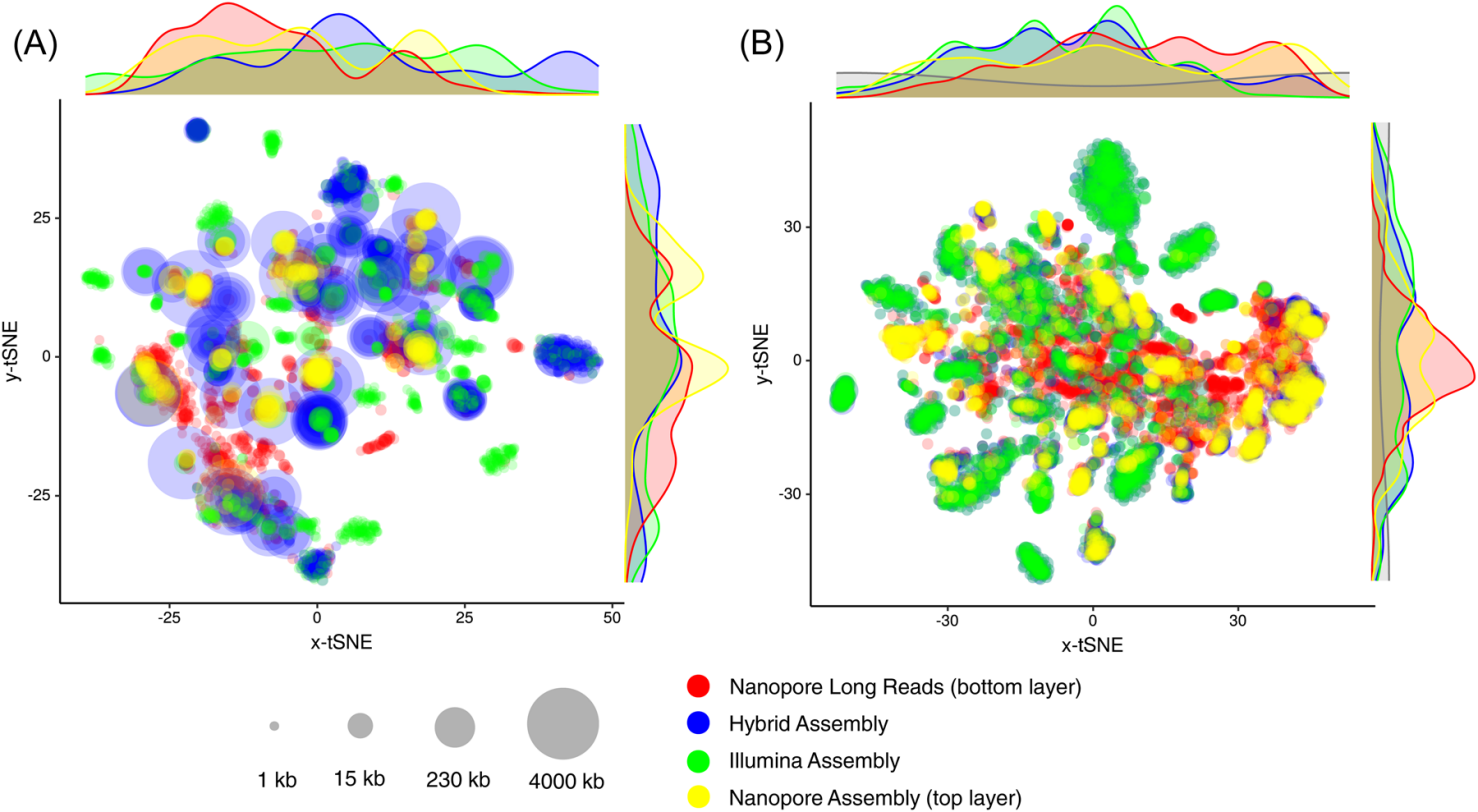
The *t*‐distributed stochastic neighbor embedding (*t*‐SNE) analysis on the microbial community of anammox bioreactor (A) and permafrost soil (B). Community composition covered by nanopore LRs, hybrid‐assembly, illumina assembly, and nanopore‐assembly were compared based on five‐nucleotide frequency. For reactor and permafrost community, 2000 and 5000 randomly picked LRs/contigs were shown. The density plots depict the density of LRs/contigs shown in the *x*‐ and *y*‐axes of the *t*‐SNE plot. LR, long read.

Another alternative for integrated metagenomic data analysis is the assembly‐free “gene‐centric” approach in which functional diversity of a community was identified and quantified directly based on the corrected nanopore LRs instead of assembled MAGs. Despite the sexiness to get long circular contigs resembling the complete bacterial genomes, a large proportion of nanopore LRs in a metagenomic data set cannot be assembled due to low coverage of the corresponding microbial population, thus being excluded from the assembly‐based “genome‐centric” analysis. As illustrated in the *t*‐distributed stochastic neighbor embedding plot, several of the condense clusters of LRs that are not covered by either hybrid‐assembled nor illumina‐alone contigs, were observed in microbiota of a partial‐nitrification anammox reactor [12] (Figure 2A). The proportion of unassembled nanopore LRs would get even higher in the permafrost community (Figure 2B) as the community diversity increases. Therefore, such a pattern is the norm rather than an exception for most natural communities with highly ununiformed community structure. Such assembly bottleneck represents a major, if not the most severe, challenge to fulfill the power of metagenomics in fully understanding community behavior and metabolic capacities of a microbiome. Given the comparable read length of nanopore LRs to the assembled contigs, the LRs data set itself is a precious deposit for genomic information that cannot be covered by the assembly strategy. The robust taxonomic resolution of nanopore LRs has facilitated the association of functional genes such as antibiotic resistance genes (ARGs) to their phylogenetic hosts and mobility elements [41, 72]. On the basis of the nanopore‐based ARGs identification, Che et al. were able to confirm that most of the ARGs detected in all compartments of the WWTPs were carried by plasmids rather than on ARGs carrying chromosomes [41]. And the phylogenetic spectrum of host populations identified by postcorrection LRs could be enlarged by 40% than that of the assembly‐based approaches in the permafrost community, facilitating the identification of vigorous aerobic methane oxidation by *Methylomonas*, which could serve as a bio‐filter to mitigate CH_4_ emissions from permafrost during thawing [50]. As a result, the assemble‐free technique in integrated metagenomic data mining deserves special attention because it can often reveal broader phylogenetic linkages of a community's metabolic capacities.

## TOOLS AND PIPELINES AVAILABLE FOR BIOINFORMATIC ANALYSIS OF ILLUMINA AND NANOPORE‐INTEGRATED METAGENOMICS

A systematic understanding of the bioinformatics tools is crucial to implement the analytical strategies aforementioned. The preanalysis adaptor trimming and quality control (QC) of illumina and nanopore raw sequences is of importance to ensure the reliability of subsequent assembly and annotation; however, given the maturity procedure of the QC steps, for example, FASTP [73] and Trimmomatic [74] for illumina, and porechop [75] for nanopore reads, the following properties of tools introduced in subsequent secessions are based on their performance using post‐QC SRs and LRs by default.

### Basecalling for nanopore signals

The raw electrical signal from a nanopore sequencer needs to be first translated into a DNA sequence by basecalling. Basecalling is a computationally extensive and rather important step as it largely determines the quality of nanopore LRs. The algorithm for basecalling is undergoing active development with new versions and tools updated frequently, thereby it is helpful for the user to check and write down the version of basecaller they used before the subsequent bioinformatic procedure. An informatic evaluation of the performance of different basecallers based on genome sequencing of *Escherichia coli* could be found at https://github.com/rrwick/Basecalling-comparison.

### Tools for de novo metagenomic assembly

Among the assemblers designed for nanopore LRs‐along assembly, only metaFlye was designed specifically to address metagenomic assembly challenges, like, uneven bacterial composition and intraspecies heterogeneity. Both Miniasm's simple overlap layout consensus algorithm and Wtdbg2's long‐kmer‐based fuzzy Bruijn graph [76] were not intended for metagenomic assembly. Due to their relatively strict and fixed coverage requirements, only the most dominant community populations could be assembled. At a sequencing depth of 10 Gb per soil sample, these tools can generate contigs sizes as small as 5 Mb. For hybrid‐assembly of illumina SRs and nanopore LRs, MetaSPAdes provides the core algorithm in which nanopore LRs will be used to simplify the SRs‐constructed De Bruijn graph by closing gaps and resolving repeats. And Unicycler is a newly designed tool to optimize such hybrid‐assembly approach of isolated bacterial strains. By semiglobally aligning nanopore LRs to the graph constructed by SPAdes algorithm, Unicycler showed improved capabilities to recover bacteria genomes into longer contigs. However, the assembly performance deteriorated with memory issue and an extended processing time when assembly metagenomic data set of the PNA system with 112 Gb paired‐end SRs and 69.4 Gb LRs [12].

### Tools for LRs/contigs correction

There are primarily three methods for correcting errors in LR‐assembled contigs. The first is to obtain consensus reads based on LR overlaps. Racon and Medaka were well known for their ability to generate consensus sequences. Both of these tools take input of contigs, nanopore raw reads, overlaps/alignments between the reads and the contigs, and produce a set of polished contigs as output. The second strategy is to polish LRs‐assembled contigs using illumina SRs. Pilon is currently the most popular tool for such purposes, but the indel identification procedure usually presents the slowest step in the overall integrated metagenomics workflow (Figure 2). Whereas, the Polypolish showed higher polishing accuracy as well as efficiency in terms of core time [77]. The final method is the frame‐shift correction. LAST invented such frame‐shift aware alignment, which was recently added to Diamond. FUNPore and MEGAN‐LR could take alignment from LAST and DIAMOND as input, respectively, and produce frame‐shifts corrected contigs. One thing to notice is that nanopore LRs could be correct following the same procedure as LR‐assembled contigs.

### Tools for metagenomic binning of the assembled genomes

The critical step of illumina and nanopore‐integrated metagenomic data analysis is to optimize the de novo assembly strategy and carry out appropriate correction steps when necessary. Once the assembly is available, standard metagenomic binning tools could be applied to obtain highly continuous HQ‐MAGs. MetaWRAP and MetaBAT were among the most commonly used binning tools in literatures applying integrated metagenomic approach. Noteworthy, it would be a practical suggestion to take the extreme long contigs (>1 Mbp) potentially obtained from different assembly tools, directly as initial bins for subsequent binning step [13]. MetaWRAP [78] is metagenomic wrapper suite whose binning module offers a combined approach to extract MAGs by using MetaBAT2 [79], MaxBin2 [80], and CONCOCT [81] algorithms and delivers refined and dereplicated binning results. It is particularly helpful when multidimensional coverage information is available. MetaBAT2 is the most commonly applied tool when processing single integrated data set without additional coverage to assistant differential coverage binning.

### Tools for annotation of postcorrected LRs/contigs

The annotation of postcorrected LRs/contigs is straightforward. Centrifuge [82] and Kraken2 [46] were the most commonly used tool for phylogenetic annotation. One thing to note is that Centrifuge only provides community‐wide phylogenetic composition instead of taxonomic assignment for each read. Consequently, ARGpore2 [72] was designed to solve this problem by applying a MEGAN‐like Lowest Common Ancestor voting algorithm. The power of clade‐specific marker genes database of MetaPhlan [83] was also integrated into ARGpore2 to improve species‐level resolution for taxonomic annotation. Prokka is the most commonly used tool for functional annotation of MAGs or postcorrected LRs/contigs. The UniProt [84] annotation it produced could be easily assigned to the KEGG pathway by tools, like, KEGG mapper [85] (Table 3).

**Table 3:** Introduction to softwares for illumina and nanopore‐integrated metagenomics

Bioinformatic category of tools	Name of tool	Description	Reference
Nanopore‐alone assembly	Canu	Canu is a fork of the Celera Assembler, designed for noisy long reads produced by PacBio or nanopore sequencing. LR assembly of Canu runs in hierarchical steps of correct‐trim‐assembly. An *adaptive overlapping strategy* was applied to improve genome recovery efficiency.	[53]
	metaFlye	De novo assembler for nanopore LR specifically designed to address important LR metagenomic assembly challenges. The uneven bacterial composition was addressed by introducing a metagenome *k*‐mer selection mode in which *genomic k‐mers were selected based on a per‐read frequency threshold* estimated based on error probability other than uniformed coverage threshold, while the intraspecies (strain‐level) heterogeneity was resolved by iteratively identifying the repetitive edges based on read‐path of the *repeat graph*.	[16]
	Miniasm	Miniasm is a very fast overlap layout consensus (OLC)‐based de novo assembler of noisy nanopore LRs. It takes all‐versus‐all LRs self‐mappings as input and generates an assembly graph in GFA format. Different from mainstream assemblers, Miniasm does not have a consensus step. It simply concatenates pieces of read sequences to generate the final contig, therefore the per‐base error rate of contigs is similar to the raw input LRs.	[52]
		It is *not specifically optimized for metagenome assembly*, therefore only the very dominant populations within a community could be assembled.	
	Wtdbg2	De novo assembler for noisy PacBio and nanopore LRs. It assembles raw LRs without error correction and then builds the consensus from intermediated assembly output. Wtdbg2 chops read into 1024 bp segments, merges similar segments into a vertex and connects vertices based on the segment adjacency on reads resulting in a *fuzzy Bruijn graph* (FBG), which is akin De Bruijn graph but permits mismatches/gaps and keeps read paths when collapsing *k*‐mers.	[76]
		It is capable to assemble large genomes at speed 10 times faster than Canu, but it is *not specifically optimized for metagenome assembly*, therefore usually only the very dominant populations could be assembled.	
Hybrid‐assembly	MetaSPAdes	MetaSPAdes is a de novo assembler capable of hybrid‐assembly of illumina SRs and nanopore LRs with the classic Spades algorithm. Nanopore LRs will be used to simplify the SR‐constructed De Bruijn graph by closing gaps and resolving repeats. MetaSPAdes will not correct the errors on nanopore LRs. The postcorrected nanopore LRs can be simply provided as single long reads to SPAdes.	[48]
	Unicycler	Unicycler is a de novo assembler designed to optimize the hybrid assembler of illumina SRs and nanopore LRs for bacterial isolates. To simplify the graph and produce longer contigs, nanopore LRs were semiglobally aligned to the assembly graph constructed based on SRs by SPAdes. If only nanopore LRs were provided as input, it will run a miniasm + Racon pipeline.	[49]
LRs‐correction	Medaka	Medaka is a tool to create consensus sequences and variant calls from nanopore sequencing data. It performs the task by neural networks, which apply a pileup of individual sequencing reads against a draft assembly.	https://github.com/nanoporetech/medaka
	Racon	Racon is intended as a standalone graph‐based consensus module to correct raw contigs generated by rapid assembly of nanopore LRs.	[57]
SRs‐correction	Pilon	Pilon is a software tool which can be used to correct indels and single base errors in nanopore data sets based on the BAM files of illumina SRs aligned to nanopore LRs.	[58]
	Polypolish	Polypolish is a tool for polishing genome assemblies with SRs, in which it uses SAM files where each read has been aligned to all possible locations (not just a single best location). This allows it to repair errors in repeat regions that other alignment‐based polishers cannot fix.	[77]
Frame‐shift correction	LAST + FUNpore	LAST is the first alignment tool to perform the frame‐shift aware alignment when aligning nucleotide sequences against a functional gene database consisting of amino acid sequences. The adaptive seed algorithm of LAST has shown the highest sensitivity in function gene identification on nanopore LR [86].	[50, 87]
		FUNpore is a software toolkit to correct the frame‐shift errors by inserting Ns into the nanopore LRs to maintain the frame based on the locations of frame‐shifts reported in the LAST alignments.	
	Diamond + MEGAN‐LR	Diamond is a widely used fast alignment tool originally designed for SR alignment. Since DIAMOND v 0.9.23, it updated with the function to perform frame‐shift aware DNA‐to‐protein alignment.	[62]
		MEGAN‐LR was a GUI‐based software which can correct frame‐shift errors in nanopore LRs. MEGAN‐LR is included in the default package of the free community version of MEGAN6.	
Alignment	LAST	LAST is a software that adopted an adaptive seed and fitting algorithm, which was ideal for DNA‐to‐DNA or DNA‐to‐protein alignment of error‐prone nanopore LRs. LAST has shown the highest sensitivity in function gene identification on nanopore LR [86].	[63]
	Minimap2	Minimap2 is a versatile sequence alignment program that aligns DNA or mRNA sequences against a large reference database. Typical use cases include: (1) mapping PacBio or nanopore reads to the human genome; (2) finding overlaps between long reads with error rate up to ~15%; (3) splice‐aware alignment of PacBio Iso‐Seq or nanopore cDNA or Direct RNA reads against a reference genome; (4) aligning illumina single‐ or paired‐end reads; (5) assembly‐to‐assembly alignment; (6) full‐genome alignment between two closely related species with divergence below ~15%.	[59]
Metagenomic binning tools	MetaWRAP	MetaWRAP is an easy‐to‐use metagenomic wrapper suit that accomplishes the core tasks of metagenomic analysis including binning, taxonomic profiling, and functional annotation. It extracts MAGs from metagenomic data sets by combining results from MetaBAT2, MaxBin2, and CONCOCT. It could deliver refined and dereplicated binning results for subsequent annotation. It is particularly useful to carry out differential binning in metagenomic data sets.	[78]
	MetaBAT2	MaxBin 2.0 employs an Expectation–Maximization (EM) algorithm to recover draft genomes from metagenomes. It is the most commonly used tool when binning single integrated metagenomic data set.	[79]
Phylogenetic annotation	Centrifuge	Centrifuge is a very rapid and memory‐efficient system for the classification of DNA sequences from microbial samples. The system uses a novel indexing scheme based on the Burrows‐Wheeler transform (BWT) and the Ferragina–Manzini (FM) index, optimized specifically for the metagenomic classification problem. Centrifuge requires a relatively small index (e.g., 4.3 GB for ~4100 bacterial genomes) yet provides a very fast classification speed.	[82]
	Kraken2	Kraken is a system for assigning taxonomic labels to short DNA sequences, usually obtained through metagenomic studies. Kraken aims to achieve high sensitivity and high speed by utilizing exact alignments of *k*‐mers and a novel classification algorithm. Kraken's accuracy is comparable with Megablast, with slightly lower sensitivity and very high precision.	[46]
	ARGpore2	ARGpore2 is a software package in which a MEGAN‐like LCA voting algorithm was first applied to generate taxonomic affiliation of each nanopore LR based on the annotation results of Centrifuge. Next, the derived affiliation will be validated and improved by LAST against MetaPhlan2 marker gene database, whose unique clade‐specific marker genes could achieve species‐level resolution for bacteria, archaea, eukaryotes, and viruses identification. This tool also annotates antibiotic resistance genes on nanopore LRs by LAST against an nt‐version of SARG database [88].	[72]
Functional annotation	Prokka	Prokka is a tool to annotate bacterial, archaeal, and viral genomes quickly and produce standards‐compliant output files. Whole genome annotation is the process of identifying features of interest in a set of genomic DNA sequences, and labeling them with useful information.	[67]

Abbreviations: GFA, graphical fragment assembly; LR, long read; SR, short read.

## CONCLUSION

In this review, we discussed the complete workflow for illumina and nanopore‐integrated metagenomic microbiome investigation. Despite ongoing algorithmic and computational challenges, such an integrated approach still presents the most robust strategy for facilitating metagenomic assembly and improving genomic resolution in deciphering functionalities of a complicated environmental microbiota. Numerous researchers have successfully utilized this integrated approach to obtain nonfragmented and well‐polished near‐finished MAGs or broaden the metabolic capacity spectrum in complex microbiomes. The analytical procedure and bioinformatic tools covered in this review may address application concerns in this fast‐developing field. However, we have to admit that future advancements in the per‐base accuracy of nanopore LRs may enable Q50 LRs‐alone assembly, further altering the ever‐changing landscape of metagenomic investigation. Sereika et al. have already shown that nanopore LRs derived from R10.4 chemistry can generate near‐finished bacterial genomes without the assistance of illumina SRs [71]. Additionally, in September 2022, Illumina Inc. presented the performance of its high‐performance LRs assays, dubbed illumina complete LR. This assay could produce data with an N50 of 6–7 kb with a compound statistic of precision and recall of 99.87%. These pieces of evidence taken together are demonstrating a predictable LRs‐alone future for genetic sequencing of biological investigations, including metagenomic studies. Currently, the major constrain of LRs‐alone microbiome research is the insufficient coverage required to ensure the effective assembly of a community with an unevenly distributed microbial composition. To overcome this bottleneck, technology advancement on the per‐base accuracy, such as further improved chemistry for nanopore sequencing and associated base calling algorithms, is crucial in addition to the development of a novel de novo assembly algorithm specifically optimized for metagenomic characteristics. Moreover, novel nanopore sequencing protocols, such as the ReadUntil method, could be applied to enhance sufficient sequencing depth of rare populations within a microbiota by selectively rejecting reads from the dominant microbes. Lastly, the gradually decreasing cost would be another factor that would expedite LRs‐alone metagenomic landscape.

## AUTHOR CONTRIBUTIONS

**Xia Yu**: Conceptualization, writing—original draft, writing—reviewing and editing, supervision, and funding acquisition. **Li Xiang**: Funding acquisition and writing—reviewing and editing. **Wu Ziqi**: Investigation and writing—original draft. **Nie Cailong**: Writing—original draft. **Cheng Zhanwen**: Formal analysis and visualization. **Sun Yuhong**: Data curation and writing—original draft. **Liu Lei**: Writing—reviewing and editing. **Zhang Tong**: Writing—reviewing and editing.

## CONFLICT OF INTEREST

The authors declare no conflict of interest.

## Data Availability

Data used for the comparison on nanopore, illumina, and hybrid‐assembly were retrieved from two of the authors' previous published research work (DOI of 10.1002/imt2.24 and 10.1186/s40168‐020‐00937‐3). Supplementary materials (figures, tables, scripts, graphical abstracts, slides, videos, Chinese translated versions and updated materials) may be found in the online DOI or iMeta Science http://www.imeta.science/.
